# Some new QEC MDS codes with large minimum distance

**DOI:** 10.1038/s41598-025-06092-9

**Published:** 2025-07-01

**Authors:** Lanqiang Li, Fuyin Tian, Ziwen Cao, Li Liu

**Affiliations:** 1 School of Information and Artificial Intelligence, Anhui Agricultural University, Hefei, 230036 Anhui China; 2 School of Mathematics, HeFei University of Technology, Hefei, 230009 Anhui China

**Keywords:** Quantum Error-Correcting codes, Maximum Distance Separable codes, Hermitian self-orthogonal, Generalized Reed–Solomon codes, Applied mathematics, Information technology

## Abstract

The advancement of Quantum Error-Correcting (QEC) Maximum Distance Separable (MDS) codes holds substantial importance in practical applications, substantially augmenting the reliability and efficiency of quantum communication and computing. This paper introduces two new classes of QEC MDS codes, which are devised through the utilization of generalized Reed–Solomon (GRS) codes and the Hermitian construction approach. The novelty of our QEC MDS codes lies in their parameters being distinct from all previously reported codes. Moreover, most of our codes possess a considerably greater minimum distance in comparison to existing codes of the same length.

## Introduction

Quantum Error-Correcting (QEC) codes play a pivotal role in the fields of quantum information processing and quantum computation^1,2^. The construction of QEC codes with better parameters has always been an important research topic. Calderbank et al. were the trailblazers in constructing certain QEC codes by capitalizing on classical linear codes. In their work^3^, they forged a crucial connection between classical linear codes and QEC codes. Leveraging Calderbank’s method, researchers have developed a plethora of binary QEC codes^4,5^ with the aid of classical linear codes. Subsequently, the research on non-binary QEC codes has also captured the significant attention of the scientific community. References^6–10^ serve as evidence of this burgeoning interest.

For any $[[n,k,d]]_q$ QEC code, its parameters have to meet the following quantum Singleton bound. When the equality $k=n-2d+2$ holds, the code is categorized as a Quantum Error-Correcting Maximum Distance Separable (QEC MDS) code.

### Lemma 1

(^6,7,9^ Quantum Singleton Bound) *Each* $[[n,k,d]]_q$ *quantum code must satisfy*

$$k\le n-2d+2.$$

Just like the classic error-correcting code, QEC MDS codes possess the optimal error-detecting and error-correcting capabilities in the context of quantum communication and computation. Constructing QEC MDS codes with a larger minimum distance *d* has long been a significant challenge in the field. It is well-established that the construction problem of QEC MDS codes with a length *n* less than or equal to $q+1$ has been comprehensively resolved in^11–13^. Nevertheless, when *n* exceeds $q+1$ and *d* is greater than $\frac{q+1}{2}$, the task of constructing QEC MDS codes remains formidable.

In recent years, notable progress has been made in the construction of QEC MDS codes for the range $q+1<n\le q^2+2$. For instance, when *n* takes on values such as $q^2\pm 1,q^2,\frac{q^2\pm 1}{2}$ and the minimum distance $d>\frac{q+1}{2}$, several QEC MDS codes have been successfully derived, as presented in^12,14–18^. Moreover, researchers have constructed QEC MDS codes with various flexible lengths through different methods. For example, graph theory has been employed in^8^, cyclic codes in^19,20^, pseudo-cyclic codes in^21^, constacyclic codes in^15,22–24^, and (extended) generalized Reed–Solomon (GRS) codes in^25–39^. In the work of Li et al.^25^ introduced an efficient approach for constructing QEC GRS codes. This method was further generalized by Jin et al. in the following years^26^. The papers^13,16,27^ demonstrated the construction of numerous new QEC MDS codes from GRS codes. These results strongly indicate that (extended) GRS codes are a rich and valuable resource for generating QEC MDS codes with $d>\frac{q+1}{2}$. In^28^, Zhang and Ge also successfully constructed some QEC MDS codes using GRS codes. Subsequently, a substantial number of QEC MDS codes with $d>\frac{q+1}{2}$ were constructed based on GRS codes, as reported in^29–36^. More recently, in Guo et al.^37^ developed some QEC MDS codes with a larger *d* and fewer restrictions on the choice of *n*. Additionally, in Jin et al.^38^ obtained a plethora of new QEC MDS codes with $d>\frac{q+1}{2}$ by concatenating two existing QEC MDS codes. This concatenation approach represents an efficient method for constructing new QEC codes. Building on this, Fang et al.^39^ also managed to obtain a significant number of new QEC MDS codes.

Prior to 2014, the majority of QEC MDS codes were constructed with a minimum distance $d\le \frac{q+1}{2}$. Only in a few cases were codes with $d>\frac{q+1}{2}$ developed, and these were often for rather limited and specific lengths. However, nowadays, there has been a notable surge in the development of QEC MDS codes with a $d>\frac{q+1}{2}$ and values approaching *q*. Inspired by this, in this work, we present two new classes of QEC MDS codes based on Hermitian self-orthogonal GRS codes, contributing to this growing body of research. Our main results are:

(1): For $q\equiv -1~(\text {mod }4)$, $\gamma \mid 2(q-1)$ and $\gamma \nmid (q-1)$, we construct $[[(\mu +1)\frac{q^2-1}{\gamma },(\mu +1)\frac{q^2-1}{\gamma }-2k,k+1]]_q$ codes, where $0\le \mu \le \frac{\gamma }{4}-1$ and $1\le k \le (\frac{\gamma +4}{8}+\mu )\frac{2(q-1)}{\gamma }$ (Theorem 8).
(2): For even $\gamma \mid (q-1)$ and $\gamma \equiv 2(\text {mod }4)$, we derive $[[(2\mu +1)\frac{q^2-1}{\gamma },(2\mu +1)\frac{q^2-1}{\gamma }-2k,k+1]]_q$ codes, where $0\le \mu \le \frac{\gamma -2}{4}$ and $1\le k \le \frac{q+1}{2}+(\mu +1)\frac{q+1}{\gamma }-2$ (Theorem 12).

The rest of the paper is organized as follows. Section “Preliminaries” introduces the definitions of GRS codes, QEC codes, and related research achievements. Section “Constructions of new QEC MDS codes” constructs the two new classes of QEC MDS codes. Finally, Section “Conclusion” summarizes the entire paper.

## Preliminaries

For an arbitrary linear code *C*, its Hermitian dual code $C^{\perp H}$ is defined in the manner:

$$C^{\perp H}=\{ \varvec{x}\in F_{q^2}^{n} : \langle \varvec{x,y}\rangle _{H}=0, \forall ~\varvec{ y}\in C\},$$

where $\langle \varvec{x,y}\rangle _{H}=\mathop {\sum }\nolimits _{i=1}^{n}x_iy_i^q$. Moreover, when the code *C* is a subset of $C^{\perp H}$, the code *C* is termed a Hermitian self-orthogonal code. Hermitian self-orthogonality is crucial in coding theory, particularly for constructing QEC codes, as it ensures effective quantum error correction.

Let $F_{q^2}[x]_k$ represent the set of all polynomials in $F_{q^2}[x]$ whose degree does not exceed $k-1$. For each *i* such that $1\le i\le n$, consider elements $\alpha _i, \upsilon _i\in F_{q^2}$. Under the condition that $\alpha _i\ne \alpha _j$ for $i\ne j$ and $\upsilon _i\ne 0$ for all *i*, we define two vectors $\varvec{ a}=(\alpha _1,\alpha _2,...,\alpha _n)$ and $\varvec{v}=(\upsilon _1,\upsilon _2,...,\upsilon _n)$. Based on these vectors $\varvec{a}$ and $\varvec{v}$, we can define a GRS code over $F_{q^2}$ as shown below:

$$GRS_k(\varvec{ a,v})=\{(\upsilon _1f(\alpha _1),\upsilon _2f(\alpha _2),...,\upsilon _nf(\alpha _n)):f(x)\in F_{q^2}[x]_k\}.$$

The code $GRS_k(\varvec{a,v})$ is an $[n,k,n-k+1]$ linear MDS code over $F_{q^2}$. Its generator matrix *G* can be explicitly written as:

$$\begin{aligned} G=\left( \begin{array}{ccccc} \upsilon _1\alpha _1^0 & \upsilon _2\alpha _2^0& \cdots & \upsilon _n\alpha _n^0\\ \upsilon _1\alpha _1^1 & \upsilon _2\alpha _2^1 & \cdots & \upsilon _n\alpha _n^1\\ \vdots & \vdots & \ddots & \vdots \\ \upsilon _1\alpha _1^{k-1} & \upsilon _2\alpha _2^{k-1} & \cdots & \upsilon _n\alpha _n^{k-1}\\ \end{array} \right) . \end{aligned}$$

It is a well-established fact that the Hermitian dual of $GRS_k(\varvec{a,v})$ is also a GRS code, and it has parameters $[n,n-k,k+1]$. The subsequent lemma provides an efficient approach for constructing Hermitian self-orthogonal GRS codes. This method will be frequently employed in the subsequent sections of this paper.

### Lemma 2

(^28^) *Let* $\varvec{a}$ *and* $\varvec{v}$ *represent the vectors as previously described. Then, we know that the code* $GRS_k(\varvec{a,v})$ *is Hermitian self-orthogonal if and only if (iff) the Euclidean inner product of* $\varvec{a}^{qi_1+i_2}$ *and* $\varvec{v}^{q+1}$ *is equal to 0 for all* $0\le i_1,i_2\le k-1$.

Furthermore, when $\alpha _i\ne 0$ for all *i*, we can represent the linear code generated by the following matrix as $C_{k,k_1}$.

$${\begin{matrix} & G_{k,k_1}(\varvec{a,v})= \begin{pmatrix} \upsilon _1\alpha _1^{k_1} & \upsilon _2\alpha _2^{k_1} & \cdots & \upsilon _n\alpha _n^{k_1} \\ \upsilon _1\alpha _1^{k_1+1} & \upsilon _2\alpha _2^{k_1+1} & \cdots & \upsilon _n\alpha _n^{k_1+1} \\ \vdots & \vdots & \ddots & \vdots \\ \upsilon _1\alpha _1^{k_1+k-1} & \upsilon _2\alpha _2^{k_1+k-1} & \cdots & \upsilon _n\alpha _n^{k_1+k-1} \end{pmatrix}. \end{matrix}}$$

Denote $\varvec{ v'}=\{\upsilon _1a_1^{k_1},\upsilon _2a_2^{k_1},\ldots ,\upsilon _na_n^{k_1}\}$. Obviously, $C_{k,k_1}$ is equivalent to the code $GRS_k(\varvec{a, v'})$. Based on Lemma 2, the following lemma can be readily deduced.

### Lemma 3

*Hold the symbol as shown above. The code* $C_{k,k_1}$ *is Hermitian self-orthogonal iff the Euclidean inner product of* $\varvec{a}^{qi_1+i_2}$ *and* $\varvec{v}^{q+1}$ *is equal to 0 for all* $k_1\le i_1,i_2\le k_1+k-1$.

Now, let us embark on an in-depth exploration of the definition of QEC codes and some associated results. Initially, consider the Hilbert space $\mathbb {C}^{q^n}$, which can be expressed as the tensor product $\mathbb {C}^{q} \otimes \mathbb {C}^{q} \otimes \cdots \otimes \mathbb {C}^{q}$ with a dimension of $q^n$. Any subspace $\mathbb {H}$ within this Hilbert space $\mathbb {C}^{q^n}$ can be designated as a QEC code. Specifically, if $\mathbb {H}$ is a $q^k$-dimensional subspace, it can be represented as an $[[n,k,d]]_q$ QEC code. Here, the parameter *d* represents the code’s error-detection and error-correction capabilities and is known as the minimum distance. More precisely, a QEC code has the ability to detect any error pattern with a weight less than $d-1$ and can correct up to $\lfloor \frac{d-1}{2} \rfloor$ errors. It is evident that the construction of QEC codes with a substantial *d* value is a pivotal aspect of quantum coding theory.

Next, we introduce a well-known construction method for QEC codes, namely the Hermitian construction.

### Lemma 4

(^7^) *If* *C* *is an* $[n,k,d]_{q^2}$ *classical linear code and* $C^{\perp H}$ *is contained in* *C*, *then there necessarily exists an* $[[n,2k-n,\ge d]]_q$ *QEC code.*

Based on this construction method, when *C* is an MDS code and $C \subseteq C^{\perp H}$, it is straightforward to deduce the following conclusion, which demonstrates the connection between QEC MDS codes and classical MDS codes.

### Lemma 5

*If* *C* *is an* $[n,k,d]_{q^2}$ *classical linear MDS code and is contained in* $C^{\perp h}$, *then there must exist an* $[[n,n-2k,k+1]]_q$ *QEC MDS code.*

## Constructions of new QEC MDS codes

We consistently assume that *q* represents an odd prime power and that $\gamma$ is a positive integer serving as a divisor of $q^2-1$. We define *m* such that $m=\frac{q^2-1}{\gamma }$. Let the multiplicative group $F_{q^2}^{*}$ be generated by the element $\omega$, that is, $F_{q^2}^{*}=\langle \omega \rangle$. Subsequently, we set $\theta =\omega ^s$. As a result, the subgroup $\langle \theta \rangle$ is contained within $F_{q^2}^{*}$ and has an order of *m*. Moreover, it can be readily verified that the cosets $\omega \langle \theta \rangle , \omega ^2\langle \theta \rangle ,\ldots ,\omega ^{\gamma }\langle \theta \rangle$ constitute all the distinct cosets of the subgroup $\langle \theta \rangle$ within the group $F_{q^2}^{*}$.

### Construction I

We posit the condition that $q\equiv -1~(\text {mod}~4)$ and take into account an integer $\gamma$ with the property that $\gamma$ divides $2(q-1)$ yet does not divide $(q-1)$. It is evident that since $q\equiv -1~(\text {mod }4)$, we have $4 \nmid (q-1)$. These circumstances entail that 4 is a divisor of $\gamma$ and that $\frac{\gamma }{4}$ is an odd integer. Moreover, we are able to infer that $8\mid (\gamma -4)$. In the subsequent discussion, we will expound upon the procedure for constructing QEC MDS codes with a length of $n=\mu \frac{q^2-1}{\gamma }$, where $\mu$ is constrained such that $1 \le \mu \le \frac{\gamma }{4}$. To accomplish this, we will utilize the following lemmas.

#### Lemma 6

*Assume* $q\equiv -1~(\text {mod}~4)$, *and* $\gamma$ *divides* $2(q-1)$ *but not* $(q-1)$. *For integers* $0\le i_1,i_2 \le q-1$, *the relation* $\frac{q^2-1}{\gamma }\mid (qi_1+i_2+\frac{q+1}{4})$ *holds iff*

$$\begin{aligned}&(i_1,i_2)=\left\{ {{\begin{array}{ll} {\Bigg (t_1\frac{2(q-1)}{\gamma }-1,t_1\frac{2(q-1)}{\gamma }+\frac{3q-1}{4}\Bigg ) \text { or }}\\ {\Bigg (t_1\frac{2(q-1)}{\gamma }+\frac{3q-1}{4},t_1\frac{2(q-1)}{\gamma }-1\Bigg )}, & 1\le t_1\le \frac{\gamma -4}{8},\\ {\Bigg (t_2\frac{2(q-1)}{\gamma },t_2\frac{2(q-1)}{\gamma }-\frac{q+1}{4}\Bigg ) \text { or }}\\ {\Bigg (t_2\frac{2(q-1)}{\gamma }-\frac{q+1}{4},t_2\frac{2(q-1)}{\gamma }\Bigg )}, & \frac{\gamma +4}{8}\le t_2\le \frac{\gamma }{2}. \end{array} }} \right. \end{aligned}$$

#### Proof

Observe that $0<qi_1+i_2+\frac{q+1}{4}\le q^2-1+\frac{q+1}{4}$ and $\gamma \frac{q^2-1}{\gamma }<q^2-1+\frac{q+1}{4}<(\gamma +1)\frac{q^2-1}{\gamma }$. The relation $\frac{q^2-1}{\gamma }\mid (qi_1+i_2+\frac{q+1}{4})$ implies that $qi_1+i_2+\frac{q+1}{4}=t\frac{q^2-1}{\gamma }$, where $1\le t\le \gamma$. In the following, we analyze two cases based on the parity of *t*:

**Case 1:** *t* **is even.**

Let $t=2t_1$ with $1\le t_1\le \frac{\gamma }{2}$. Then, $qi_1+i_2=2t_1\frac{q^2-1}{\gamma }-\frac{q+1}{4}$. Expanding $q^2-1=(q-1)(q+1)$, we substitute $\gamma \mid 2(q-1)$:

$$\begin{aligned} qi_1+i_2&=t_1\frac{2(q-1)}{\gamma }q+t_1\frac{2(q-1)}{\gamma }-\frac{q+1}{4}. \end{aligned}$$

Recombining the terms of *q*:

- For $$1\le t_1\le \frac{\gamma -4}{8}$$, $$-q<t_1\frac{2(q-1)}{\gamma }-\frac{q+1}{4}<0$$, so
  $$\begin{aligned} qi_1+i_2&=\Bigg (t_1\frac{2(q-1)}{\gamma }-1\Bigg )q+\Bigg (q+t_1\frac{2(q-1)}{\gamma }-\frac{q+1}{4}\Bigg )\nonumber \\&= \Bigg (t_1\frac{2(q-1)}{\gamma }-1\Bigg )q+\Bigg (t_1\frac{2(q-1)}{\gamma }+\frac{3q-1}{4}\Bigg ).~~~ \end{aligned}$$
  Note that $0<t_1\frac{2(q-1)}{\gamma }-1,t_1\frac{2(q-1)}{\gamma }+\frac{3q-1}{4}<q$. This gives $(i_1,i_2)=\big (t_1\frac{2(q-1)}{\gamma }-1,t_1\frac{2(q-1)}{\gamma }+\frac{3q-1}{4}\big )$.
- For $$\frac{\gamma +4}{8}\le t_1\le \frac{\gamma }{2}$$, $$0<t_1\frac{2(q-1)}{\gamma },t_1\frac{2(q-1)}{\gamma }-\frac{q+1}{4}<q$$, so
  $$\begin{aligned} qi_1+i_2&=\Bigg (t_1\frac{2(q-1)}{\gamma }\Bigg )q+\Bigg (t_1\frac{2(q-1)}{\gamma }-\frac{q+1}{4}\Bigg ). \end{aligned}$$
  This gives $$(i_1,i_2)=\big (t_1\frac{2(q-1)}{\gamma },t_1\frac{2(q-1)}{\gamma }-\frac{q+1}{4}\big )$$.

**Case 2:** *t* **is odd.**

Let $t=2t_1+1$ with $0\le t_2\le \frac{\gamma }{2}-1$. Then:

$$\begin{aligned} qi_1+i_2&=(2t_2+1)\frac{q^2-1}{\gamma }-\frac{q+1}{4}. \end{aligned}$$

Expanding gives:

$$\begin{aligned} qi_1+i_2= &\; 2t_2\frac{q^2-1}{\gamma }+\frac{q^2-1}{\gamma }-\frac{q+1}{4}~~~~\\= &\; t_2\frac{2(q-1)}{\gamma }(q+1)+\frac{q^2-1}{\gamma }-\frac{q+1}{4}~~~~\\= &\; t_2\frac{2(q-1)}{\gamma }q+t_2\frac{2(q-1)}{\gamma }+\frac{2(q-1)}{\gamma }\frac{q+1}{2}-\frac{q+1}{4}. \end{aligned}$$

Since $\gamma \mid 2(q-1)$ but $\gamma \nmid (q-1)$, we known that $\frac{2(q-1)}{\gamma }$ is odd. Then, we have

$$\begin{aligned}&\frac{2(q-1)}{\gamma }\frac{q+1}{2}=\frac{1}{2}\Bigg (\frac{2(q-1)}{\gamma }-1\Bigg )q+\frac{1}{2}\Bigg (\frac{2(q-1)}{\gamma }+q\Bigg ). \end{aligned}$$

Furthermore, we get

$$\begin{aligned} qi_1+i_2&= t_2\frac{2(q-1)}{\gamma }q+t_2\frac{2(q-1)}{\gamma }+\frac{1}{2}\Bigg (\frac{2(q-1)}{\gamma }-1\Bigg )q+\frac{1}{2}\Bigg (\frac{2(q-1)}{\gamma }+q\Bigg )-\frac{q+1}{4}~~~~~\\&= \Bigg (t_2\frac{2(q-1)}{\gamma }+\frac{1}{2}\Bigg (\frac{2(q-1)}{\gamma }-1\Bigg )\Bigg )q+\Bigg (t_2\frac{2(q-1)}{\gamma }+\frac{1}{2}\Bigg (\frac{2(q-1)}{\gamma }+q\Bigg )-\frac{q+1}{4}\Bigg ). \end{aligned}$$

Recombining the terms of *q*:

- For $$0\le t_2\le \frac{3(\gamma -4)}{8}+1$$, $$0<t_2\frac{2(q-1)}{\gamma }+\frac{1}{2}\big (\frac{2(q-1)}{\gamma }+q\big )-\frac{q+1}{4}<q$$, so
  $$\begin{aligned}qi_1+i_2&= \Bigg (t_2\frac{2(q-1)}{\gamma }+\frac{1}{2}\Bigg (\frac{2(q-1)}{\gamma }-1\Bigg )\Bigg )q+\Bigg (t_2\frac{2(q-1)}{\gamma }+\frac{1}{2}\Bigg (\frac{2(q-1)}{\gamma }-1\Bigg )+\frac{q+1}{4}{\Bigg )}.\end{aligned}$$
  This gives $(i_1,i_2)=\big (t_2\frac{2(q-1)}{\gamma }+\frac{1}{2}\big (\frac{2(q-1)}{\gamma }-1\big ),t_2\frac{2(q-1)}{\gamma }+\frac{1}{2}\big (\frac{2(q-1)}{\gamma }-1\big )+\frac{q+1}{4}\big )$. Now, we let $t_2=t^{'}-\frac{\gamma +4}{8}$ with $\frac{\gamma +4}{8}\le t^{'}\le \frac{\gamma }{2}$. Substituting $t_2$ into the expression of $(i_1,i_2)$ and simplifying through algebraic operations, we obtain $(i_1,i_2) =(t^{'}\frac{2(q-1)}{\gamma }-\frac{q+1}{4},t^{'}\frac{2(q-1)}{\gamma })$.
- For $$\frac{3(\gamma -4)}{8}+2\le t_2\le \frac{\gamma }{2}-1$$, $$q<t_2\frac{2(q-1)}{\gamma }+\frac{1}{2}{\big (}\frac{2(q-1)}{\gamma }+q{\big )}-\frac{q+1}{4}<2q$$, so
  $$\begin{aligned}qi_1+i_2&= {\Bigg (}t_2\frac{2(q-1)}{\gamma }+\frac{1}{2}{\Bigg (}\frac{2(q-1)}{\gamma }-1{\Bigg )}+1{\Bigg )}q+{\Bigg (}t_2\frac{2(q-1)}{\gamma }+\frac{1}{2}{\Bigg (}\frac{2(q-1)}{\gamma }-1{\Bigg )}+\frac{q+1}{4}-q{\Bigg )}\\&= {\Bigg (}t_2\frac{2(q-1)}{\gamma }+\frac{1}{2}{\Bigg (}\frac{2(q-1)}{\gamma }+1{\Bigg )}{\Bigg )}q+{\Bigg (}t_2\frac{2(q-1)}{\gamma }+\frac{1}{2}{\Bigg (}\frac{2(q-1)}{\gamma }-1{\Bigg )}-\frac{3q-1}{4}{\Bigg )}.~~~~~~~~\end{aligned}$$
  This gives $(i_1,i_2)=\big (t_2\frac{2(q-1)}{\gamma }+\frac{1}{2}{\big (}\frac{2(q-1)}{\gamma }+1\big ),t_2\frac{2(q-1)}{\gamma }+\frac{1}{2}{\big (}\frac{2(q-1)}{\gamma }-1{\big )}-\frac{3q-1}{4}\big )$. Now, we let $t_2=\frac{3(\gamma -4)}{8}+1+t^{'}$ with $1\le t^{'}\le \frac{\gamma -4}{8}$. Similarly, we have $(i_1,i_2) =(t^{'}\frac{2(q-1)}{\gamma }+\frac{3q-1}{4},t^{'}\frac{2(q-1)}{\gamma }-1)$.

Thus, we have obtained the desired results for the pairs $(i_1,i_2)$. $\square$

#### Lemma 7

*Keep the definitions of* *q* *and* $\gamma$ *consistent with Lemma* 6. *Suppose* $\mu$ *is an integer satisfying* $1\le \mu \le \frac{\gamma }{4}-1$ *and* $0\le i_1,i_2\le (\frac{\gamma +4}{8}+\mu )\frac{2(q-1)}{\gamma }-1$. *If* $\frac{q^2-1}{\gamma }$ *divides* $(qi_1+i_2+\frac{q+1}{4})$, *then the following system of equations*

1 $$\begin{aligned}&\mathop {\sum }\limits _{t=1}^{\mu +1}\beta _t^{qi_1+i_2}u_t=0, \end{aligned}$$

*admits a solution* $(u_1,u_2,\dots ,u_{\mu +1})\in (F_q^{*})^{\mu +1}$, *where* $\beta _t=\omega ^{4t}$ *for all* $1\le t\le \mu +1$.

#### Proof

Given that $1\le \mu \le \frac{\gamma }{4}-1$, we can establish that $(\frac{\gamma +4}{8}+\mu )\frac{2(q-1)}{\gamma }-1<\frac{3q-1}{4}$. For all pairs of integers $i_1$ and $i_2$ such that $0\le i_1,i_2\le (\frac{\gamma +4}{8}+\mu )\frac{2(q-1)}{\gamma }-1$, we can leverage Lemma 6. This lemma enables us to show that $\frac{q^2-1}{\gamma }\mid (qi_1+i_2+\frac{q+1}{4})$ if and only if $(i_1,i_2)$ takes one of the following forms: $(t_2\frac{2(q-1)}{\gamma },t_2\frac{2(q-1)}{\gamma }-\frac{q+1}{4})$ or $(t_2\frac{2(q-1)}{\gamma }-\frac{q+1}{4},t_2\frac{2(q-1)}{\gamma })$, where $\frac{\gamma +4}{8}\le t_2\le \frac{\gamma +4}{8}+\mu -1$. In other words, we can express $qi_1+i_2$ as either $2t_2\frac{(q^2-1)}{\gamma }-\frac{q+1}{4}$ or $2t_2\frac{(q^2-1)}{\gamma }-\frac{q^2-1}{4}-\frac{q+1}{4}$. To simplify the notation, we let $t_2=\frac{\gamma +4}{8}+j$, where $0\le j\le \mu -1$. Then, $qi_1+i_2$ can be rewritten as $\frac{q^2-1}{4}+(2j+1)\frac{(q^2-1)}{\gamma }-\frac{q+1}{4}$ or $(2j+1)\frac{(q^2-1)}{\gamma }-\frac{q+1}{4}$ for $0\le j\le \mu -1$. We now define two matrices. Let

$$\begin{aligned} \varvec{A}=\left( \begin{array}{ccccc} \beta _1^{\frac{q^2-1}{\gamma }-\frac{q+1}{4}}& \beta _2^{\frac{q^2-1}{\gamma }-\frac{q+1}{4}} & \cdots & \beta _{\mu +1}^{\frac{q^2-1}{\gamma }-\frac{q+1}{4}}\\ \beta _1^{\frac{q^2-1}{4}+\frac{q^2-1}{\gamma }-\frac{q+1}{4}}& \beta _2^{\frac{q^2-1}{4}+\frac{q^2-1}{\gamma }-\frac{q+1}{4}} & \cdots & \beta _{\mu +1}^{\frac{q^2-1}{4}+\frac{q^2-1}{\gamma }-\frac{q+1}{4}}\\ \vdots & \vdots & \ddots & \vdots \\ \beta _1^{(2\mu -1)\frac{q^2-1}{\gamma }-\frac{q+1}{4}}& \beta _2^{(2\mu -1)\frac{q^2-1}{\gamma }-\frac{q+1}{4}} & \cdots & \beta _{\mu +1}^{(2\mu -1)\frac{q^2-1}{\gamma }-\frac{q+1}{4}}\\ \beta _1^{\frac{q^2-1}{4}+(2\mu -1)\frac{q^2-1}{\gamma }-\frac{q+1}{4}}& \beta _2^{\frac{q^2-1}{4}+(2\mu -1)\frac{q^2-1}{\gamma }-\frac{q+1}{4}} & \cdots & \beta _{\mu +1}^{\frac{q^2-1}{4}+(2\mu -1)\frac{q^2-1}{\gamma }-\frac{q+1}{4}}\\ \end{array} \right) , \end{aligned}$$

and

$$\begin{aligned} \varvec{A^{'}}=\left( \begin{array}{ccccc} \beta _1^{\frac{q^2-1}{\gamma }}& \beta _2^{\frac{q^2-1}{\gamma }} & \cdots & \beta _{\mu +1}^{\frac{q^2-1}{\gamma }}\\ \beta _1^{3\frac{q^2-1}{\gamma }}& \beta _2^{3\frac{q^2-1}{\gamma }} & \cdots & \beta _{\mu +1}^{3\frac{q^2-1}{\gamma }}\\ \vdots & \vdots & \ddots & \vdots \\ \beta _1^{(2\mu -1)\frac{(q^2-1)}{\gamma }}& \beta _2^{(2\mu -1)\frac{(q^2-1)}{\gamma }} & \cdots & \beta _{\mu +1}^{(2\mu -1)\frac{(q^2-1)}{\gamma }}\\ \end{array} \right) . \end{aligned}$$

Evidently, the system of equations (1) can be written in matrix form as

$$\begin{aligned} \varvec{A}\left( \begin{array}{c} x_1 \\ x_2 \\ \vdots \\ x_{\mu +1}\\ \end{array} \right) =\varvec{0}. \end{aligned}$$

Recall that for any $\beta _t=\omega ^{4t}$, we have $\beta _t^{\frac{q^2-1}{4}}=1$ and $\beta _t^{\frac{q^2-1}{4}+(2j+1)\frac{(q^2-1)}{\gamma }-\frac{q+1}{4}}= \beta _t^{(2j+1)\frac{(q^2-1)}{\gamma }-\frac{q+1}{4}}$. Leveraging these equalities, we can further simplify the above system to

2 $$\begin{aligned} \varvec{A^{'}}\left( \begin{array}{c} \beta _1^{-\frac{q+1}{4}}x_1 \\ \beta _2^{-\frac{q+1}{4}}x_2 \\ \vdots \\ \beta _{\mu +1}^{-\frac{q+1}{4}}x_{\mu +1}\\ \end{array} \right) =\varvec{A^{'}}\left( \begin{array}{c} y_1 \\ y_2 \\ \vdots \\ y_{\mu +1}\\ \end{array} \right) =\textbf{0}, \end{aligned}$$

where $y_t=\beta _t^{-\frac{q+1}{4}}x_t$ for all $1\le t\le \mu +1$. Since $\beta _t=\omega ^{4t}$, we can show that $\beta _t^{\frac{q^2-1}{\gamma }}=\omega ^{2t\frac{2(q-1)}{\gamma }(q+1)}\in F_q^{*}$ for all $1\le t\le \mu +1$. This property implies that every entry of $\varvec{A^{'}}$ belongs to $F_q^{*}$, which means $\varvec{A^{'q}}=\varvec{A^{'}}$. Moreover, it is clear that any $\mu$ columns of $\varvec{A^{'}}$ are linearly independent. Notice that $\varvec{A^{'}}$ is an $\mu \times (\mu +1)$ matrix. According to $[31, \text {Lemma 2.3}]$, the system of equations (2) has a non-zero solution $(y_1,y_2,\dots ,y_{\mu +1})$ in $(F_q^{*})^{\mu +1}$. Let $u_t=y_t\beta _t^{\frac{q+1}{4}}$ for all $1\le t\le \mu +1$. Since $\beta _t^{\frac{q+1}{4}}=\omega ^{t(q+1)}\in F_q^{*}$, it follows that $(u_1,u_2,\dots ,u_{\mu +1})\in (F_q^{*})^{\mu +1}$ is a non-zero solution of (1). $\square$

Let $\mu$ be an integer satisfying $0\le \mu \le \frac{\gamma }{4}-1$. Define $\beta _t=\omega ^{4t}$ for each *t* in the range $1\le t\le \mu +1$. Recall that the value of *m* is defined as $m=\frac{q^2-1}{\gamma }$. At this point, consider the vectors $\varvec{ a}$ and $\varvec{ v}$ defined as follows:

$$\varvec{ a}=(\beta _1,\beta _1\theta ,\ldots ,\beta _1\theta ^{m-1},\ldots ,\beta _{\mu +1},\beta _{\mu +1}\theta , \ldots ,\beta _{\mu +1}\theta ^{m-1})\in F_{q^2}^{m(\mu +1)},$$

$$\varvec{ v}=(v_1,v_1\omega ^{\frac{\gamma }{4}},\ldots ,v_1\omega ^{\frac{\gamma }{4}(m-1)},\ldots ,v_{\mu +1},v_{\mu +1}\omega ^{\frac{\gamma }{4}},\ldots ,v_{\mu +1}\omega ^{\frac{\gamma }{4}(m-1)})\in (F_{q^2}^{*})^{m(\mu +1)},$$

where $v_1,v_2,\ldots ,v_{\mu +1}$ are elements of $F_{q^2}^{*}$. By leveraging the code $GRS_k(\varvec{a,v})$, we are in a position to derive the following theorem.

#### Theorem 8

*We assume that* $q\equiv -1~(\text {mod}~4)$*and take into account an integer* $\gamma$ *with the property that* $\gamma$ *divides* $2(q-1)$ *yet does not divide* $(q-1)$. *Let* $\mu$ *be a fixed integer such that* $0\le \mu \le \frac{\gamma }{4}-1$. *For every* *k* *in the range* $1\le k \le (\frac{\gamma +4}{8}+\mu )\frac{2(q-1)}{\gamma }$, *a* $[[(\mu +1)\frac{q^2-1}{\gamma },(\mu +1)\frac{q^2-1}{\gamma }-2k,k+1]]_q$ *QEC MDS code can be constructed.*

#### Proof

To begin with, we calculate the following expression:

$$\begin{aligned} \langle \varvec{a}^{qi_1+i_2},\varvec{ v}^{q+1}\rangle _E= & \sum _{t=1}^{\mu +1}\beta _t^{qi_1+i_2}v_t^{q+1}\sum _{l=0}^{m-1}\theta ^{(qi_1+i_2+\frac{q+1}{4})l}. \end{aligned}$$

It is important to note that the sum $\mathop {\sum }\nolimits _{l=0}^{m-1}\theta ^{(qi_1+i_2+\frac{q+1}{4})l}$ has the following behavior:

$$\begin{aligned} \sum ^{m-1}_{l=0}\theta ^{(qi_1+i_2+\frac{q+1}{4})l}=\left\{ {{\begin{array}{ll} {0}, & m \nmid \left( qi_1+i_2+\frac{q+1}{4}\right) ,\\ \\ {m}, & m\mid \left( qi_1+i_2+\frac{q+1}{4}\right) .\\ \end{array} }} \right. \end{aligned}$$

Consequently, the value of $\langle \varvec{a}^{qi_1+i_2},\varvec{v}^{q+1}\rangle _E$ can be expressed as:

$$\begin{aligned} \langle \varvec{a}^{qi_1+i_2},\varvec{v}^{q+1}\rangle _E=\left\{ {{\begin{array}{ll} {0}, & m \nmid \left( qi_1+i_2+\frac{q+1}{4}\right) ,\\ \\ {m\mathop {\sum }\limits _{t=1}^{\mu +1}\beta _t^{qi_1+i_2}v_t^{q+1}}, & m\mid \left( qi_1+i_2+\frac{q+1}{4}\right) .\\ \end{array} }} \right. \end{aligned}$$

When $\mu =0$, by referring to Lemma 6, we can conclude that $m \nmid (qi_1+i_2+\frac{q+1}{4})$ for any $0\le i_1,i_2\le (\frac{\gamma +4}{8}+\mu )\frac{2(q-1)}{\gamma }-1$, i.e.,

$$\begin{aligned} \langle \varvec{a}^{qi_1+i_2},\varvec{v}^{q+1}\rangle _E=0. \end{aligned}$$

When $1\le \mu \le \frac{\gamma }{4}-1$, we can take $v_t^{q+1}=u_t$ for all $1\le t\le \mu +1$, where $u_t\in F_q^{*}$ is obtained from Lemma 7. According to Lemma 7, we have $\mathop {\sum }\nolimits _{t=1}^{\mu +1}\beta _t^{qi_1+i_2}v_t^{q+1}=\mathop {\sum }\nolimits _{t=1}^{\mu +1}\beta _t^{qi_1+i_2}u_t=0$, when $m\mid (qi_1+i_2+\frac{q+1}{4})$. In summary, for all pairs of integers $i_1$ and $i_2$ such that $0\le i_1,i_2\le k-1$, the value of $\langle \varvec{a}^{qi_1+i_2},\varvec{v}^{q+1}\rangle _E$ is equal to 0. According to Lemma 2, the code $GRS_k(\varvec{a,v})$ is a Hermitian self-orthogonal code. Additionally, it is straightforward to see that $GRS_k(\varvec{a,v})$ is a $[(\mu +1)\frac{q^2-1}{\gamma },k,(\mu +1)\frac{q^2-1}{\gamma }-k+1]$ MDS code. Finally, by applying Lemma 5, we can construct a $[[(\mu +1)\frac{q^2-1}{\gamma },(\mu +1)\frac{q^2-1}{\gamma }-2k,k+1]]_q$ QEC MDS code. $\square$

When $\gamma \ne 4$, by substituting $\mu =\frac{\gamma }{4}-1$ into Theorem 8, the following QEC MDS code can be readily derived. This corollary further expands the scope of QEC MDS code construction, providing a specific case that can be directly applied under the given conditions.

#### Corollary 9

*Maintain the same definitions of* *q* *and* $\gamma$ *as in Theorem* 8. *For all values of* *k* *such that* $1\le k \le \frac{3(q-1)}{4}-\frac{q-1}{\gamma }$, *a* $[[\frac{q^2-1}{4},\frac{q^2-1}{4}-2k,k+1]]_q$ *QEC MDS code can be constructed.*

In Table 1, we display a collection of novel QEC MDS codes that have been derived from Theorem 8 and Corollary 9. Notably, each of these codes is characterized by a parameter *d* that exceeds the value of $\frac{q+1}{2}$. This particular feature endows these codes with enhanced error-correction capabilities, setting them apart and making them of significant interest within the domain of quantum error correction research and application.

**Table 1:** New QEC MDS codes derived from construction I.

*q*	$\gamma$	$\mu$	QEC code	*k*
$10\mid (q-1)$	20	4	$[[\frac{q^2-1}{4},\frac{q^2-1}{4}-2k,k+1]]_q$	$1\le k\le \frac{7(q-1)}{10}$
$14\mid (q-1)$	28	5	$[[\frac{3(q^2-1)}{14},\frac{3(q^2-1)}{14}-2k,k+1]]_q$	$1\le k\le \frac{9(q-1)}{14}$
$18\mid (q-1)$	36	7	$[[\frac{2(q^2-1)}{9},\frac{2(q^2-1)}{9}-2k,k+1]]_q$	$1\le k\le \frac{2(q-1)}{3}$
$22\mid (q-1)$	44	9	$[[\frac{5(q^2-1)}{22},\frac{5(q^2-1)}{22}-2k,k+1]]_q$	$1\le k\le \frac{15(q-1)}{22}$
$26\mid (q-1)$	52	11	$[[\frac{3(q^2-1)}{13},\frac{3(q^2-1)}{13}-2k,k+1]]_q$	$1\le k\le \frac{9(q-1)}{13}$

#### Remark 1

Within the framework of Theorem 8, by specifying the range of the parameter $\mu$ such that $\frac{\gamma -4}{8}\le \mu \le \frac{\gamma }{4}-1$, the QEC MDS codes we have constructed possess a minimum distance *d* that exceeds $\frac{q+1}{2}$. As the value of $\gamma$ asymptotically approaches $2(q-1)$ and $\mu$ approaches $\frac{\gamma }{4}$, through verification, it can be demonstrated that the expression $(\frac{\gamma +4}{8}+\mu )\frac{2(q-1)}{\gamma }+1$ approaches $\frac{3q}{4}$. This outcome indicates that our codes can attain a parameter *d* approaching $\frac{3q}{4}$, while the lengths of these codes only approach $\frac{q^2-1}{4}$. Such characteristics endow our codes with potentially enhanced error-correcting capabilities and favorable length-distance trade-offs, which are of particular significance in the context of quantum error correction and quantum information processing.

In the context of Theorem 8, a particular class of QEC MDS codes has been devised, characterized by the parameters $[[(\mu +1)\frac{q^2-1}{\gamma },(\mu +1)\frac{q^2-1}{\gamma }-2k,k+1]]_q$. Here, the parameters are constrained such that $0\le \mu \le \frac{\gamma }{4}-1$ and $1\le k \le (\frac{\gamma +4}{8}+\mu )\frac{2(q-1)}{\gamma }$.

In the case where $\mu +1$ is odd, the length of our codes can be formulated as

$$n=(\mu +1)\frac{2(q-1)}{\gamma }\frac{q+1}{2}.$$

It is pertinent to note that $\frac{2(q-1)}{\gamma }$ is an odd quantity. Consequently, the length *n* of our codes represents an odd multiple of $\frac{q+1}{2}$. This implies that the length *n* is not divisible by either $q+1$ or $q-1$. In comparison to the previously known QEC MDS codes, our code exhibits a novel and distinct length characteristic, which sets it apart in the realm of quantum error correction.

When $\mu +1$ is an even integer, the length of our codes can be expressed as

$$n=\frac{(\mu +1)(q-1)}{\gamma }(q+1).$$

Generally, it can be observed that $\frac{(\mu +1)(q-1)}{\gamma }$ does not act as a divisor of $q-1$. Subsequently, we posit the condition $\lambda \nmid (q-1)$ and proceed to conduct a comparative analysis between our codes and the QEC MDS codes with parameters $[[\lambda (q+1),\lambda (q+1)-2k,k+1]]_q$ that have been documented in the existing literature. This comparison aims to further elucidate the unique properties and potential advantages of our proposed codes within the broader landscape of QEC codes.

In the studies presented in Theorem 3.5 of^17^ by Shi et al. and Theorem 6 of^37^ by Guo et al., a specific class of QEC MDS codes has been constructed. These codes are characterized by the parameters $[[\lambda (q+1),\lambda (q+1)-2k,k+1]]_q$, where the parameters are further constrained such that $1\le \lambda \le q-1$ and $1\le k\le \lambda -1$. By setting $\lambda =\frac{(\mu +1)(q-1)}{\gamma }$, it can be observed that these QEC MDS codes possess the same length as our codes, specifically $\frac{(\mu +1)(q-1)}{\gamma }(q+1)$. However, in terms of the minimum distances, the codes constructed by Shi et al. and Guo et al. have $d\le \lambda$, which in this case is equivalent to $\frac{(\mu +1)(q-1)}{\gamma }$. It is worth highlighting that the inequality $\frac{(\mu +1)(q-1)}{\gamma }<(\frac{\gamma +4}{8}+\mu )\frac{2(q-1)}{\gamma }+1$ holds. This clearly indicates that our codes are endowed with a significantly larger minimum distance.

In the research detailed in Theorem 3.2 of^28^ by Zhang et al., a particular class of QEC MDS codeshas been derived. These codes are defined by the parameters $[[b\frac{q-1}{2a}(q+1),b\frac{q-1}{2a}(q+1)-2k,k+1]]_q$, with the conditions that 2*a* divides $(q-1)$, $1\le b\le 2a$ and $1\le k\le \frac{q-1}{2}+\frac{q-1}{2a}$. By setting $b=2(\mu +1)$ and $2a=\frac{\gamma }{2}$, it is evident that the length of these QEC MDS codes is also $\frac{(\mu +1)(q-1)}{\gamma }(q+1)$. However, in terms of the minimum distance, these codes have $d\le \frac{q-1}{2}+\frac{2(q-1)}{\gamma }+1$. It is important to note that when $\frac{\gamma +4}{8}\le \mu \le \frac{\gamma }{4}-1$, the inequality $\frac{q-1}{2}+\frac{2(q-1)}{\gamma }+1\le (\frac{\gamma +4}{8}+\mu )\frac{2(q-1)}{\gamma }+1$ holds. This conclusively demonstrates that our codes possess a substantially larger minimum distance.

In the works of Theorem 4.12 of^29^ by Shi et al. and Corollary 3.4 of^32^ by Fang et al., a class of QEC MDS codes codes has been constructed. These codes are parameterized as $[[\frac{\theta (q-1)}{h}(q+1),\frac{\theta (q-1)}{h}(q+1)-2k,k+1]]_q$, under the conditions that *h* divides $(q-1)$, $1\le \theta \le h$ and $1\le k\le \frac{\theta (q-1)}{h}-1$. By setting $h=\frac{\gamma }{2}$ and $\theta =\frac{\mu +1}{2}$, it can be observed that their QEC MDS codes possess a length of $\theta \frac{q^2-1}{h}=(\mu +1)\frac{q^2-1}{\gamma }$, which coincides with the length of our codes described in Theorem 8. Nevertheless, in terms of the minimum distance, their codes have $d\le \frac{\theta (q-1)}{h}=\frac{(\mu +1)(q-1)}{\gamma }$. It is noteworthy that the inequality $\frac{(\mu +1)(q-1)}{\gamma }< (\frac{\gamma +4}{8}+\mu )\frac{2(q-1)}{\gamma }$ holds. This clearly indicates that our codes exhibit a significantly larger minimum distance.

The above discussion theoretically indicates that the minimum distance of most of our codes is larger compared with the existing codes of the same length. Now, Table 2 provides specific parameter comparisons with prior literature (e.g.,^17,28,32,37^) in order to more intuitively quantify the claimed advantage of “larger minimum distance”.

**Table 2:** QEC MDS codes with parameters $[[n,n-2d+2,d]]_{q}$.

*q*	$\gamma$	$\mu$	*n*	*d* in Construction I	*d* in^17,29,32,37^	*d* in^28^
11	20	3	24	$2 \le d \le 10$	$d=2$	$2 \le d \le 7$
23	44	7	96	$2 \le d \le 14$	$2 \le d \le 4$	$2 \le d \le 13$
23	44	9	108	$2 \le d \le 16$	$2 \le d \le 5$	$2 \le d \le 13$
31	20	3	192	$2 \le d \le 19$	$2 \le d \le 6$	$2 \le d \le 19$
43	28	5	396	$2 \le d \le 28$	$2 \le d \le 9$	$2 \le d \le 25$
51	20	3	520	$2 \le d \le 31$	$2 \le d \le 10$	$2 \le d \le 31$
63	124	17	576	$2 \le d \le 34$	$2 \le d \le 9$	$2 \le d \le 33$
63	124	19	640	$2 \le d \le 36$	$2 \le d \le 10$	$2 \le d \le 33$
63	124	25	832	$2 \le d \le 42$	$2 \le d \le 13$	$2 \le d \le 33$
63	124	29	960	$2 \le d \le 46$	$2 \le d \le 15$	$2 \le d \le 33$
67	44	7	816	$2 \le d \le 40$	$2 \le d \le 12$	$2 \le d \le 37$
67	44	9	1020	$2 \le d \le 46$	$2 \le d \le 15$	$2 \le d \le 37$

### Construction II

We consider the scenario where $\gamma$ is an even divisor of $q+1$ and $\gamma \equiv 2(\text {mod 4})$. Next, we will detail the procedure for constructing QEC MDS codes. These codes have a length of $n=(2\mu +1)\frac{q^2-1}{\gamma }$, with the parameter $\mu$ satisfying the constraint $0\le \mu \le \frac{\gamma -2}{4}$. To achieve this construction, the following lemmas are indispensable.

#### Lemma 10

*Assume* $\gamma$ *is an even divisor of* $q+1$ *with* $\gamma \equiv 2(\text {mod 4})$. *For integers* $0\le i_1,i_2 \le q-1$, *the relation* $\frac{q^2-1}{\gamma }\mid {\big (}qi_1+i_2+\frac{q+1}{2}{\big )}$ *holds iff*

$$\begin{aligned}&(i_1,i_2)=\left\{ {{\begin{array}{ll} {{\Bigg (}l_1\frac{q+1}{\gamma }-1,\frac{q+1}{2}-l_1\frac{q+1}{\gamma }-1{\Bigg )}\text { or }}\\ {{\Bigg (}\frac{q+1}{2}-l_1\frac{q+1}{\gamma }-1,l_1\frac{q+1}{\gamma }-1{\Bigg )}}, & 1\le l_1\le \frac{\gamma -2}{4},\\ {{\Bigg (}l_2\frac{q+1}{\gamma }-2,\frac{3(q+1)}{2}-l_2\frac{q+1}{\gamma }-2{\Bigg )} \text { or }}\\ {{\Bigg (}\frac{3(q+1)}{2}-l_2\frac{q+1}{\gamma }-2,l_2\frac{q+1}{\gamma }-2{\Bigg )}}, & \frac{\gamma }{2}\le l_2\le \frac{3\gamma -2}{4}. \end{array} }} \right. \end{aligned}$$

#### Proof

First, note that the inequality $0<qi_1+i_2+\frac{q+1}{2}\le q^2-1+\frac{q+1}{2}$ holds, and we also have $\gamma \frac{q^2-1}{\gamma }<q^2-1+\frac{q+1}{2}<(\gamma +1)\frac{q^2-1}{\gamma }$. The relation $\frac{q^2-1}{\gamma }\mid (qi_1+i_2+\frac{q+1}{4})$ implies: $qi_1+i_2+\frac{q+1}{2}=l\frac{q^2-1}{\gamma }$, where $1\le l\le \gamma$. Since $\gamma \equiv 2(\text {mod 4})$, we know that $4|(\gamma -2)$. In the following, we analyze two intervals for *l*:

**Case 1:** $$1\le l\le \frac{\gamma }{2}-1$$.

In this case, we can express $qi_1+i_2$ as:

$$\begin{aligned} qi_1+i_2&=l\frac{q+1}{\gamma }q-l\frac{q+1}{\gamma }-\frac{q+1}{2}~~~~~~~~~~~~~~~~~\\&={\Bigg (}l\frac{q+1}{\gamma }-1{\Bigg )}q+{\Bigg (}\frac{q+1}{2}-l\frac{q+1}{\gamma }-1{\Bigg )}. \end{aligned}$$

- For $1\le l\le \frac{\gamma -2}{4}$, it is clear that $0<l\frac{q+1}{\gamma }-1,$, $\frac{q+1}{2}-l\frac{q+1}{\gamma }-1<q$. Therefore, $(i_1,i_2)$ = ${\big (}l\frac{q+1}{\gamma }-1,\frac{q+1}{2}-l\frac{q+1}{\gamma }-1{\big )}$.
- For $\frac{\gamma -2}{4}+1\le l\le \frac{\gamma }{2}-1$, we let $l=\frac{\gamma }{2}-l_1$ with $1\le l_1\le \frac{\gamma -2}{4}$. Then, we can rewrite $qi_1+i_2$ as $qi_1+i_2 ={\big (}\frac{q+1}{2}-l\frac{q+1}{\gamma }-1{\big )}q+{\big (}l\frac{q+1}{\gamma }-1{\big )}$. Therefore, $(i_1,i_2)={\big (}\frac{q+1}{2}-l_1\frac{q+1}{\gamma }-1,l_1\frac{q+1}{\gamma }-1{\big )}$.

**Case 2:** $$\frac{\gamma }{2}\le l\le \gamma$$.

Note that $-q<\frac{q+1}{2}-l\frac{q+1}{\gamma }-1<0$. In this case, the expression for $qi_1+i_2$ becomes:

$$\begin{aligned} qi_1+i_2&={\Bigg (}l\frac{q+1}{\gamma }-2{\Bigg )}q+{\Bigg (}q+\frac{q+1}{2}-l\frac{q+1}{\gamma }-1{\Bigg )}\\&={\Bigg (}l\frac{q+1}{\gamma }-2{\Bigg )}q+{\Bigg (}\frac{3(q+1)}{2}-l\frac{q+1}{\gamma }-2{\Bigg )}. \end{aligned}$$

Given that , in this case, $(i_1,i_2)= (l\frac{q+1}{\gamma }-2,\frac{3(q+1)}{2}-l\frac{q+1}{\gamma }-2)$ with $\frac{\gamma }{2}\le l\le \gamma$. More precisely, when . Subsequently, when , let $l=\frac{3\gamma }{2}-l_2$, where . Then, we obtain $(i_1,i_2)=(\frac{3(q+1)}{2}-l_2\frac{q+1}{\gamma }-2,l_2\frac{q+1}{\gamma }-2)$ with $\frac{\gamma }{2}\le l_2\le \frac{3\gamma -2}{4}$.

- For $\frac{\gamma }{2}\le l\le \frac{3\gamma -2}{4}$, it is clear that $0<l\frac{q+1}{\gamma }-2,\frac{3(q+1)}{2}-l\frac{q+1}{\gamma }-2<q$. Therefore, $(i_1,i_2)$ = $(l\frac{q+1}{\gamma }-2,\frac{3(q+1)}{2}-l\frac{q+1}{\gamma }-2)$.
- For $\frac{3\gamma -2}{4}+1\le l\le \gamma$, let $l=\frac{3\gamma }{2}-l_2$ with $\frac{\gamma }{2}\le l_2\le \frac{3\gamma -2}{4}$. Then, we can rewrite $qi_1+i_2$ = ${\big (}\frac{3(q+1)}{2}-l_2\frac{q+1}{\gamma }-2{\big )}q+{\big (}l_2\frac{q+1}{\gamma }-2{\big )}$. Therefore, $(i_1,i_2)$ = ${\big (}\frac{3(q+1)}{2}-l_2\frac{q+1}{\gamma }-2,l_2\frac{q+1}{\gamma }-2 {\big )}$.

Through the above-detailed analysis, we have successfully proven this lemma. $\square$

#### Lemma 11

*Keep the definitions of* *q* *and* $\gamma$ *consistent with Lemma* 10. *Suppose that* $1\le \mu \le \frac{\gamma -2}{4}$, *and consider integers* $i_1$ *and* $i_2$ *within the range* $\frac{\gamma -2}{4}\frac{q+1}{\gamma }\le i_1,i_2\le \frac{3(q+1)}{2}-(\frac{3\gamma -2}{4}-\mu )\frac{q+1}{\gamma }-3$. *If* $\frac{q^2-1}{\gamma }$ *divides* $(qi_1+i_2+\frac{q+1}{2})$, *then the following system of equations*

3 $$\begin{aligned} \sum _{t=1}^{2\mu +1}\beta _t^{qi_1+i_2}u_t=0 \end{aligned}$$

*admits a solution* $(u_1,u_2,\dots ,u_{2\mu +1})\in (F_q^{*})^{2\mu +1}$, *where* $\beta _t=\omega ^{2t}$ *for all* $1\le t\le 2\mu +1$.

#### Proof

We start by observing the inequality $\frac{\gamma -2}{4}\frac{q+1}{\gamma }\le i_1,i_2\le \frac{3(q+1)}{2}-(\frac{3\gamma -2}{4}-\mu )$ $\frac{q+1}{\gamma }-3$. Relying on Lemma 10, the condition $\frac{q^2-1}{\gamma }\mid (qi_1+i_2-\frac{q+1}{2})$ is satisfied if and only if $(i_1,i_2)$ takes the form $(l_2\frac{q+1}{\gamma }-2,\frac{3(q+1)}{2}-l_2\frac{q+1}{\gamma }-2)$ or $(\frac{3(q+1)}{2}-l_2\frac{q+1}{\gamma }-2,l_2\frac{q+1}{\gamma }-2)$, where $\frac{3\gamma -2}{4}-\mu +1\le l_2\le \frac{3\gamma -2}{4}$. In other words, we can express $qi_1+i_2$ as either $l_2\frac{(q^2-1)}{\gamma }-\frac{q+1}{2}$ or $\frac{3(q^2-1)}{2}-l_2\frac{(q^2-1)}{\gamma }-\frac{q+1}{2}$. We also note that $\frac{3(q^2-1)}{2}-l_2\frac{(q^2-1)}{\gamma }-\frac{q+1}{2}$ = $(\frac{3\gamma }{2}-l_2)\frac{(q^2-1)}{\gamma }-\frac{q+1}{2}$. By denoting $l^{'}=\frac{3\gamma }{2}-l_2$, we obtain that $\frac{3\gamma +2}{4}\le l^{'}\le \frac{3\gamma +2}{4}+\mu -1$. Consequently, $qi_1+i_2=l_2\frac{(q^2-1)}{\gamma }-\frac{q+1}{2}$ or $l^{'}\frac{(q^2-1)}{\gamma }-\frac{q+1}{2}$, where $\frac{3\gamma -2}{4}-\mu +1\le l_2\le \frac{3\gamma -2}{4}$ and $\frac{3\gamma +2}{4}\le l^{'}\le \frac{3\gamma +2}{4}+\mu -1$. That is, $qi_1+i_2=l\frac{(q^2-1)}{\gamma }-\frac{q+1}{2}$ with $\frac{3\gamma -2}{4}-\mu +1\le l\le \frac{3\gamma +2}{4}+\mu -1$. For the sake of convenience, we define the following two matrices. Let

$$\begin{aligned} \varvec{A}=\left( \begin{array}{ccccc} \beta _1^{(\frac{3\gamma -2}{4}-\mu +1)\frac{(q^2-1)}{\gamma }-\frac{q+1}{2}}& \beta _2^{(\frac{3\gamma -2}{4}-\mu +1)\frac{(q^2-1)}{\gamma }-\frac{q+1}{2}} & \cdots & \beta _{2\mu +1}^{(\frac{3\gamma -2}{4}-\mu +1)\frac{(q^2-1)}{\gamma }-\frac{q+1}{2}}\\ \beta _1^{(\frac{3\gamma -2}{4}-\mu +2)\frac{(q^2-1)}{\gamma }-\frac{q+1}{2}}& \beta _2^{(\frac{3\gamma -2}{4}-\mu +2)\frac{(q^2-1)}{\gamma }-\frac{q+1}{2}} & \cdots & \beta _{2\mu +1}^{(\frac{3\gamma -2}{4}-\mu +2)\frac{(q^2-1)}{\gamma }-\frac{q+1}{2}}\\ \vdots & \vdots & \ddots & \vdots \\ \beta _1^{(\frac{3\gamma +2}{4}+\mu -1)\frac{(q^2-1)}{\gamma }-\frac{q+1}{2}}& \beta _2^{(\frac{3\gamma +2}{4}+\mu -1)\frac{(q^2-1)}{\gamma }-\frac{q+1}{2}} & \cdots & \beta _{2\mu +1}^{(\frac{3\gamma +2}{4}+\mu -1)\frac{(q^2-1)}{\gamma }-\frac{q+1}{2}}\\ \end{array} \right) \end{aligned}$$

and

$$\begin{aligned} \varvec{A^{'}}=\left( \begin{array}{ccccc} \beta _1^{(\frac{3\gamma -2}{4}-\mu +1)\frac{(q^2-1)}{\gamma }}& \beta _2^{(\frac{3\gamma -2}{4}-\mu +1)\frac{(q^2-1)}{\gamma }} & \cdots & \beta _{2\mu +1}^{(\frac{3\gamma -2}{4}-\mu +1)\frac{(q^2-1)}{\gamma }}\\ \beta _1^{(\frac{3\gamma -2}{4}-\mu +2)\frac{(q^2-1)}{\gamma }}& \beta _2^{(\frac{3\gamma -2}{4}-\mu +2)\frac{(q^2-1)}{\gamma }} & \cdots & \beta _{2\mu +1}^{(\frac{3\gamma -2}{4}-\mu +2)\frac{(q^2-1)}{\gamma }}\\ \vdots & \vdots & \ddots & \vdots \\ \beta _1^{(\frac{3\gamma +2}{4}+\mu -1)\frac{(q^2-1)}{\gamma }}& \beta _2^{(\frac{3\gamma +2}{4}+\mu -1)\frac{(q^2-1)}{\gamma }} & \cdots & \beta _{2\mu +1}^{(\frac{3\gamma +2}{4}+\mu -1)\frac{(q^2-1)}{\gamma }}\\ \end{array} \right) . \end{aligned}$$

Evidently, the system of equations (3) can be represented in matrix form as

$$\begin{aligned} \varvec{A}\left( \begin{array}{c} x_1 \\ x_2 \\ \vdots \\ x_{2\mu +1}\\ \end{array} \right) =\varvec{0}. \end{aligned}$$

This system can be further simplified to

4 $$\begin{aligned} \varvec{A^{'}}\left( \begin{array}{c} \beta _1^{-\frac{q+1}{2}}x_1 \\ \beta _2^{-\frac{q+1}{2}}x_2 \\ \vdots \\ \beta _{2\mu +1}^{-\frac{q+1}{2}}x_{2\mu +1}\\ \end{array} \right) =\varvec{A^{'}}\left( \begin{array}{c} y_1 \\ y_2 \\ \vdots \\ y_{2\mu +1}\\ \end{array} \right) =\varvec{0}, \end{aligned}$$

where $y_t=\beta _t^{-\frac{q+1}{2}}x_t$ for all $1\le t\le 2\mu +1$. Under the operation modulo $q^2-1$, we derive that

$$\begin{aligned} l_2\frac{q^2-1}{\gamma }q= & l_2\frac{q+1}{\gamma }q-l_2\frac{q+1}{\gamma }q\\= & {\Bigg (}(l_2\frac{q+1}{\gamma }-2)q+\frac{3(q+1)}{2}-l_2\frac{q+1}{\gamma }-2+\frac{q+1}{2}{\Bigg )}q\\= & {\Bigg (}\frac{3(q+1)}{2}-l_2\frac{q+1}{\gamma }-2{\Bigg )}q+{\Bigg (}l_2\frac{q+1}{\gamma }-2+\frac{q+1}{2}{\Bigg )}\\= & {\Bigg (}\frac{3(q+1)}{2}q+\frac{q+1}{2}-2(q+1){\Bigg )}-{\Bigg (}l_2\frac{q+1}{\gamma }q-l_2\frac{q+1}{\gamma }{\Bigg )}\\= & {\Bigg (}\frac{3q}{2}+\frac{1}{2}-2{\Bigg )}(q+1)-l_2\frac{q+1}{\gamma }(q-1)\\= & \frac{3\gamma }{2}\frac{q^2-1}{\gamma }-l_2\frac{q^2-1}{\gamma }\\= & {\Bigg (}\frac{3\gamma }{2}-l_2{\Bigg )}\frac{q^2-1}{\gamma }. \end{aligned}$$

Recall that $l^{'}=\frac{3\gamma }{2}-l_2$, we obtain $l_2\frac{q^2-1}{\gamma }q=l^{'}\frac{q^2-1}{\gamma }$. That is, $\beta _t^{l_2\frac{q^2-1}{\gamma }q}=\beta _t^{l^{'}\frac{q^2-1}{\gamma }}$ with $\frac{3\gamma -2}{4}-\mu +1$ $\le l_2\le \frac{3\gamma -2}{4}$ and $\frac{3\gamma +2}{4}\le l^{'}\le \frac{3\gamma +2}{4}+\mu -1$. This implies that $A^{'}$ is row equivalent to $\varvec{A^{'q}}$, i.e., $\varvec{A^{'q}}=\varvec{A^{'}}$. Moreover, it is evident that $A^{'}$ is an $2\mu \times (2\mu +1)$ matrix and any $2\mu$ columns are linearly independent. According to $[31, \text {Lemma 2.3}]$, the system of equations (4) has a non-zero solution $(y_1,y_2,\dots ,y_{2\mu +1})$ in $(F_q^{*})^{2\mu +1}$. Letting $u_t=y_t\beta _t^{\frac{q+1}{2}}$ for all $1\le t\le 2\mu +1$. Since $\beta _t^{\frac{q+1}{2}}\in F_q^{*}$ (as $\beta _t\in T_e$), we conclude that $(u_1,u_2,\dots ,u_{2\mu +1})\in (F_q^{*})^{2\mu +1}$ is a non-zero solution of (3). $\square$

Let $\mu$ be an integer such that $1\le \mu \le \frac{\gamma -2}{4}$. Define $\beta _t=\omega ^{2t}$ for each *t* in the range $1\le t\le 2\mu +1$. And recall that $m=\frac{q^2-1}{\gamma }$. Consider the vectors:

$$\varvec{ a}=(\beta _1,\beta _1\theta ,\dots ,\beta _1\theta ^{m-1},\dots ,\beta _{2\mu +1},\beta _{2\mu +1}\theta ,\dots ,\beta _{2\mu +1}\theta ^{m-1}),$$

$$\varvec{ v}=(v_1,v_1\omega ^{\frac{\gamma }{2}},\ldots ,v_1\omega ^{\frac{\gamma }{2}(m-1)},\dots ,v_{2\mu +1},v_{2\mu +1}\omega ^{\frac{\gamma }{2}},\ldots ,v_{2\mu +1}\omega ^{\frac{\gamma }{2}(m-1)}),$$

where $v_1,v_2,\ldots ,v_{2\mu +1}$ are elements of $F_{q^2}^{*}$. By leveraging the code $C_{k,k_1}(\varvec{a,v})$, we can derive the following theorem.

#### Theorem 12

*Assume that* $\gamma$ *is an even divisor of* $q+1$ *and* $\gamma \equiv 2(\text {mod 4})$. *Fix* $\mu$, $0\le \mu \le \frac{\gamma -2}{4}$. *For every integer* *k* within the range $1\le k \le \frac{q+1}{2}+(\mu +1)\frac{q+1}{\gamma }-2$, *a* $[[(2\mu +1)\frac{q^2-1}{\gamma },(2\mu +1)\frac{q^2-1}{\gamma }-2k,k+1]]_q$ *QEC MDS code can be constructed.*

#### Proof

Set $k_1=\frac{\gamma -2}{4}\frac{q+1}{\gamma }$. Next, we consider the GRS code $C_{k,k_1}$. When $\mu =0$, we get

$$\begin{aligned} \langle \varvec{a}^{qi_1+i_2},\varvec{ v}^{q+1}\rangle _E= & \beta _1^{qi_1+i_2}v_1^{q+1}\sum _{l=0}^{m-1}\theta ^{(qi_1+i_2+ \frac{q+1}{2})l}. \end{aligned}$$

We note that the sum $\sum ^{m-1}_{l=0}\theta ^{(qi_1+i_2+\frac{q+1}{2})l}$ has the following property:

$$\begin{aligned} \sum ^{m-1}_{l=0}\theta ^{(qi_1+i_2+\frac{q+1}{2})l}=\left\{ {{\begin{array}{ll} {0}, & m\nmid \left( qi_1+i_2+\frac{q+1}{2}\right) ,\\ \\ {m}, & m\mid \left( qi_1+i_2+\frac{q+1}{2}\right) .\\ \end{array} }} \right. \end{aligned}$$

From Lemma 10, we know that for all $i_1$ and $i_2$ in the range $k_1\le i_1,i_2\le k_1+\frac{q+1}{2}+\frac{q+1}{\gamma }-2-1$, the relation $m \nmid (qi_1+i_2+\frac{q+1}{2})$ holds. When $1\le k \le \frac{q+1}{2}+\frac{q+1}{\gamma }-2$, for all $i_1$ and $i_2$ such that $k_1\le i_1,i_2\le k_1+k-1$, the value of $\langle \varvec{a}^{qi_1+i_2},\varvec{ v}^{q+1}\rangle _E$ is equal to 0. From Lemma 3, this implies that $C_{k,k_1}(\varvec{a, v})\subseteq C_{k,k_1}(\varvec{a, v})^{\perp _H}$ for all *k* in the range $1\le k \le \frac{q+1}{2}+\frac{q+1}{\gamma }-2$. When $1\le \mu \le \frac{\gamma -2}{4}$, we get

$$\begin{aligned} \langle \varvec{a}^{qi_1+i_2},\varvec{ v}^{q+1}\rangle _E=\sum _{t=1}^{2\mu +1}\beta _t^{qi_1+i_2}v_t^{q+1}\sum _{l=0}^{m-1}\theta ^{(qi_1+i_2+\frac{q+1}{2})l}. \end{aligned}$$

According to Lemma 11, for all $i_1$ and $i_2$ in the range $\frac{\gamma -2}{4}\frac{q+1}{\gamma }\le i_1,i_2$ $\le \frac{3(q+1)}{2}-(\frac{3\gamma -2}{4}-\mu )\frac{q+1}{\gamma }-3$, which is equivalent to $k_1\le i_1,i_2\le k_1$ $+\frac{q+1}{2}+(\mu +1)\frac{q+1}{\gamma }-2-1$, there exist a tuple $(u_1,u_2,\dots ,u_{2\mu +1})\in (F_q^{*})^{2\mu +1}$ such that $\mathop {\sum }\nolimits _{t=1}^{2\mu +1}\beta _t^{qi_1+i_2}u_t=0$. For $t=1,2,\ldots , 2\mu +1$, we choose $v_t\in F_{q^2}^{*}$ such that $v_t^{q+1}=u_t$. Then, when $1\le k \le \frac{q+1}{2}+(\mu +1)\frac{q+1}{\gamma }-2$, for all $i_1$ and $i_2$ with $k_1\le i_1,i_2\le k_1+k-1$, the value of $\langle \varvec{a}^{qi_1+i_2},\varvec{ v}^{q+1}\rangle _E$ is equal to 0. Once again, by Lemma 3, we can conclude that $C_{k,k_1}(\varvec{a, v})\subseteq C_{k,k_1}(\varvec{a, v})^{\perp _H}$ for all *k* in the range $1\le k \le \frac{q+1}{2}+(\mu +1)\frac{q+1}{\gamma }-2$.

Recall that $C_{k,k_1}(\varvec{a,v})$ is an $[n,k,n-k+1]$ MDS code. Therefore, by applying Lemma 5, we obtain the desired result. $\square$

With $\mu =0$, the following QEC MDS code can be readily derived from Theorem 12.

#### Corollary 13

*Keep the definitions of* *q* *and* $\gamma$ *consistent with Theorem* 12. *For each value of* *k* *such that* $1\le k \le \frac{q+1}{2}+\frac{q+1}{\gamma }-2$, *a* $[[\frac{q^2-1}{\gamma },\frac{q^2-1}{\gamma }-2k,k+1]]_q$ *QEC MDS codeis guaranteed to exist.*

Moreover, when we consider the specific case of $\gamma =2$ in Corollary 13, we can get the following QEC MDS code. This code is also one of the main results presented in^22^, although the methods employed in our work differ from those in the cited reference.

#### Corollary 14

*Let* *k* *be an interge satisfing* $1\le k\le q-1$. *Then, a* $[[\frac{q^2-1}{2},\frac{q^2-1}{2}+1-2k,k+1]]_q$ *QEC MDS code is certain to exist.*

In Table 3, we showcase a selection of novel QEC MDS codes that have been derived from Theorem 12. Notably, each of these codes is characterized by a parameter *d* that surpasses the value of $\frac{q+1}{2}$, thereby highlighting their potentially enhanced error-correcting capabilities and distinctiveness within the realm of quantum error correction.

**Table 3:** New QEC MDS codes derived from construction II.

*q*	$\gamma$	$\mu$	QEC code	*k*
$10\mid (q+1)$	10	1	$[[\frac{3(q^2-1)}{10},\frac{3(q^2-1)}{10}-2k,k+1]]_q$	$1\le k\le \frac{7(q+1)}{10}-2$
$14\mid (q+1)$	14	2	$[[\frac{5(q^2-1)}{14},\frac{5(q^2-1)}{14}-2k,k+1]]_q$	$1\le k\le \frac{5(q+1)}{7}-2$
$18\mid (q+1)$	18	3	$[[\frac{7(q^2-1)}{18},\frac{7(q^2-1)}{18}-2k,k+1]]_q$	$1\le k\le \frac{13(q+1)}{18}-2$
$22\mid (q+1)$	22	4	$[[\frac{9(q^2-1)}{22},\frac{9(q^2-1)}{22}-2k,k+1]]_q$	$1\le k\le \frac{8(q+1)}{11}-2$
$26\mid (q+1)$	26	5	$[[\frac{11(q^2-1)}{26},\frac{11(q^2-1)}{26}-2k,k+1]]_q$	$1\le k\le \frac{19(q+1)}{26}-2$

#### Remark 2

Theorem 12 constructed a class of QEC MDS codes, which are parameterized as $[[(2\mu +1)\frac{q+1}{\gamma }(q-1),(2\mu +1)\frac{q+1}{\gamma }(q-1)-2k,k+1]]_q$. Here, the parameters are constrained such that $0\le \mu \le \frac{\gamma -2}{4}$ and $1\le k \le \frac{q+1}{2}+(\mu +1)\frac{q+1}{\gamma }-2$. It is evident that all QEC MDS codes constructed in Theorem 12 have *d* that exceeds $\frac{q+1}{2}$. Next, we will compare our codes with the QEC MDS codes documented in the literature. Subsequently, we will undertake a comparative analysis between our codes and those QEC MDS codes that have been previously documented in the existing body of literature, with the aim of elucidating the unique characteristics and potential advantages of our proposed codes.

In Theorem 3.8 of^17^, under the condition that $q\equiv 1~(\text {mod}~4)$, a particular class of QEC MDS codes has been established. These codes are characterized by the parameters $[[2(2\delta +1)(q-1),2(2\delta +1)(q-1)-2k,k+1]]_q$ with the constraints $0\le \delta \le \frac{q-1}{4}$ and $1\le k\le 4\delta +1$. Given that $q\equiv 1~(\text {mod}~4)$, it can be inferred that $(2\mu +1)\frac{q+1}{\gamma }$ is invariably odd. In contrast, it is evident that $2(2\delta +1)$ is an even quantity. Consequently, the length of the codes described in Theorem 3.8 of^17^ diverges from that of the codes presented in our Theorem 12. Furthermore, within Theorem 4.8 of^28^, another class of QEC MDS codes has been constructed, possessing a length of $(2t+2)\frac{q^2-1}{h}=(2t+2)\frac{q+1}{h}(q-1)$, where *h* represents an even divisor of $q+1$. It is manifest that the length of these codes also differs from that of our Theorem 12, since $2\mu +1$ is an odd integer. This disparity in code lengths serves to distinguish our proposed codes from those in the aforementioned references, highlighting their unique characteristics and potential advantages in the realm of quantum error correction.

In the studies presented in Theorem 4.2 of^28^ and Corollary 5.5 of^32^, under the assumption that 2*a* divides $(q+1)$, Zhang et al. and Fang et al. successfully derived a class of QEC MDS codes, which are characterized by the parameters $[[b\frac{q+1}{2a}(q-1),b\frac{q+1}{2a}(q-1)-2k,k+1]]_q$. Here, the parameters are further constrained such that $1\le b\le 2a$ and $1\le k\le \frac{q+1}{2}+\frac{q+1}{2a}-2$. By setting $b=2\mu +1$ and $2a=\gamma$, it becomes evident that the aforementioned QEC MDS codes share the same length, namely $\frac{(2\mu +1)(q+1)}{\gamma }(q-1)$, as the codes under our investigation. Additionally, it is determined that their $d\le \frac{q+1}{2}+\frac{q+1}{\gamma }-1$. It is noteworthy that when $\mu >0$, the inequality $\frac{q+1}{2}+\frac{q+1}{\gamma }-1\le \frac{q+1}{2}+(\mu +1)\frac{q+1}{\gamma }-1$ holds. Therefore, our codes have much larger *d*. This clearly implies that our codes possess a significantly larger minimum distance.

In Theorem 6.3 of^32^, under the conditions where 2*h* divides $(q+1)$ and $1\le k\le \frac{q+1}{2}+\frac{\tau (q+1)}{2h}-2$ with $1\le \tau \le h-1$, a certain class of QEC MDS codes, denoted by the parameters $[[(2\tau +1)\frac{q^2-1}{2h},(2\tau +1)\frac{q^2-1}{2h}-2k,k+1]]_q$, has been constructed. By setting $\tau =\mu$ and $2h=\gamma$, it becomes evident that these codes possess a length of $(2\tau +1)\frac{q^2-1}{2h}=(2\mu +1)\frac{q^2-1}{\gamma }$, which coincides with the length of our codes as presented in Theorem 12. Nevertheless, through deduction, it can be ascertained that when $\tau =\mu$ and $2h=\gamma$, the inequality $\frac{q+1}{2}+\frac{\tau (q+1)}{2h}-2< \frac{q+1}{2}+(\mu +1)\frac{q+1}{\gamma }-2$ holds. This inequality clearly indicates that our codes are endowed with a substantially larger minimum distance.

In Theorem 5 of^37^, a particular class of QEC MDS codes, characterized by the parameters $[[\nu (q-1),\nu (q-1)-2k,k+1]]_q$ is derived. Here, the parameters are constrained such that $1\le \nu \le q$ and $1\le k\le \lfloor \frac{\nu q-1}{q+1}\rfloor$. By setting $\nu =\frac{(2\mu +1)(q+1)}{\gamma }$, it is observed that the codes in question possess the same length as the ones under our consideration. However, their $d\le \lfloor \frac{2\mu +1}{\gamma }q-\frac{1}{q+1}\rfloor +1$. Given the condition $0\le \mu \le \frac{\gamma -2}{4}$, it can be deduced that $\lfloor \frac{2\mu +1}{\gamma }q-\frac{1}{q+1}\rfloor +1\le \frac{q+1}{2}+\frac{q+1}{\gamma }$. This relationship conclusively demonstrates that the codes we have developed exhibit a significantly larger minimum distance.

In the following, Table 4 presents the specific parameter comparisons with previous literature (such as^28,32,37^), more intuitively demonstrating the claimed advantage of “greater minimum distance”.

**Table 4:** QEC MDS codes with parameters $[[n,n-2d+2,d]]_{q}$.

*q*	$\gamma$	$\mu$	*n*	*d* in Construction II	*d* in^28^	*d* in^32^	*d* in^35^
11	6	1	60	$2 \le d \le 9$	$2 \le d \le 7$	$2 \le d \le 7$	$2 \le d \le 6$
17	6	1	144	$2 \le d \le 14$	$2 \le d \le 11$	$2 \le d \le 11$	$2 \le d \le 9$
19	10	1	108	$2 \le d \le 13$	$2 \le d \le 11$	$2 \le d \le 11$	$2 \le d \le 6$
19	10	2	180	$2 \le d \le 15$	$2 \le d \le 11$	$2 \le d \le 13$	$2 \le d \le 10$
23	6	1	264	$2 \le d \le 19$	$2 \le d \le 15$	$2 \le d \le 15$	$2 \le d \le 12$
27	14	1	156	$2 \le d \le 17$	$2 \le d \le 15$	$2 \le d \le 15$	$2 \le d \le 6$
27	14	2	260	$2 \le d \le 19$	$2 \le d \le 15$	$2 \le d \le 17$	$2 \le d \le 10$
27	14	3	364	$2 \le d \le 21$	$2 \le d \le 15$	$2 \le d \le 19$	$2 \le d \le 14$
41	14	1	360	$2 \le d \le 26$	$2 \le d \le 23$	$2 \le d \le 23$	$2 \le d \le 9$
41	14	2	600	$2 \le d \le 29$	$2 \le d \le 23$	$2 \le d \le 26$	$2 \le d \le 15$
41	14	3	840	$2 \le d \le 32$	$2 \le d \le 23$	$2 \le d \le 29$	$2 \le d \le 21$

## Conclusion

In the present paper, two novel classes of QEC MDS codes are constructed leveraging GRS codes and the Hermitian construction methodology. For one of the newly devised code classes, the minimum distance *d* is capable of exceeding $\frac{q+1}{2}$ (See Table 1), whereas for the other class, *d* invariably surpasses this value (See Table 3). These codes possess the characteristic of having $d\ge \frac{q+1}{2}$ and asymptotically approach *q*. Such attributes are of particular significance as they augment the error correction proficiency of the codes. Moreover, a salient feature of our research endeavor is that the constructed QEC MDS codes possess parameters hitherto unreported in the extant literature (See Table 5). It has been further ascertained that the majority of the constructed codes display a substantially larger *d* in contrast to the known QEC MDS codes of equivalent length, thereby signifying their potential for enhanced error correction performance. Remarks 1 and 2 not only emphasize that the minimum distance of our codes exceeds $\frac{q+1}{2}$, but also provide theoretical analysis by comparing with the previous references^17,28,32,37^ to quantify the claimed advantage of “larger minimum distance”. Moreover, Tables 2 and 4 presented the specific parameter comparisons with prior literature. In Table 5, we summarized the parameters of some known QEC MDS codes with minimum distance $d> \frac{q+1}{2}$.

**Table 5:** Some known QEC MDS codes with minimum distance $d>\frac{q+1}{2}$.

Code length	Constraints	Minimum distances	References
$\frac{q^2-1}{2}$	*q* odd	$2\le d\le q$	^22^
$\lambda (q+1)$	$\lambda$ odd, $\lambda \mid (q-1)$	$2 \le d \le \frac{q+1}{2}+\lambda$	^22^
$2\lambda (q+1)$	$q \equiv 1 (\textrm{mod}~4)$, $\lambda$ odd, $\lambda \mid (q-1)$	$2 \le d \le \frac{q+1}{2}+2 \lambda$	^22^
$\lambda (q-1)$	$\lambda = \frac{q+1 }{r}$ with even *r*	$2 \le d \le \frac{q+1}{2}+ \lambda -1$	^22,24^
$\lambda (q-1)$	$\lambda = \frac{q+1 }{r}$ with odd *r*	$2 \le d \le \frac{q+1}{2}+ \frac{\lambda }{2}-1$	^24^
$\frac{q^2-1}{h}$	*q* odd, $h \mid (q+1)$, $h \in {{3,5,7}}$	$2 \le d \le \frac{(q+1)(h+1)}{2h}-1$	^23^
$2t(q-1)$	$8 \mid (q+1)$, $t \mid (q+1)$	$2 \le d \le 6t+1$	^23^
$3(q-1)$	$3^{2} \mid (q+1)$ with odd *q*	$2 \le d \le \frac{q+5}{2}$	^23^
$(2t+1)\frac{q^{2}-1}{2s+1}$	$(2s+1) \mid (q+1)$, $1 \le t \le s$	$2 \le d \le (s+t+1)\frac{q-1}{2s+1}-1$	^27^
$2t \frac{q^{2}-1}{2s+1}$	$(2s+1) \mid (q+1)$, $1 \le t \le s-1$	$2 \le d \le (s+t+1)\frac{q-1}{2s+1}-1$	^27,29^
$2t \frac{q^{2}-1}{2s}$	$2s \mid (q+1)$, $1 \le t \le s$	$2 \le d \le (s+t) \frac{q-1}{2s}-1$	^28,29^
$r \frac{q^{2}-1}{2s}$	$2s \mid (q+1)$, $1 \le r \le 2s$	$2 \le d \le (s+1) \frac{q-1}{2s}$	^28^
$r \frac{q^{2}-1}{s}$	$s \mid (q-1)$, $1 \le r \le s$	$2 \le d \le r \frac{q-1}{s}$	^29,32^
$(2t+1)\frac{q^{2}-1}{2s}$	$2s \mid (q-1)$, $1 \le t \le 2s$	$2 \le d \le (s+t) \frac{q-1}{2s}-1$	^32^
$\frac{q^2-1}{4}+\frac{q^2-1}{h}$	$\frac{2(q-1)}{h}=2\tau +1$	$2\le d \le \frac{q-1}{2}+\tau$	^33^
$\frac{q^2-1}{4}+\frac{2(q^2-1)}{h}$	$\frac{2(q-1)}{h}=2\tau +1$, $(h\ne 4)$	$2\le d \le \frac{q-1}{2}+2\tau +1$	^33^
$m(q-1)$	$1 \le m \le q$	$2 \le d \le \lfloor \frac{mq-1}{q+1}\rfloor +1$	^37^
$\frac{2(r_1+1)r_2(q^2-1)}{h}$	$q\equiv 3~(\text {mod}~4)$, $h=h_1h_2\ge 9$, $h_1\mid \frac{q-1}{2}$, $h_2\mid (q+1)$, $r_1\le h_1-1$, odd $h_2>2r_2$	$2 \le d \le \text {max}\{\frac{(r_1-1)(q-1)}{2h_1},$ $\frac{2(r_2-1)(q+1)}{h_2}\}+2$	^35^
$\frac{(5t+q-1)(q+1)}{9}$	$3\mid (q+1)$,$t\mid (q+1)$,$3\mid (t-1)$	$2 \le d \le \frac{q+2t-1}{3} +1$	^36^
$\frac{(7t+q-1)(q+1)}{9}$	$3\mid (q+1)$,$t\mid (q+1)$,$3\mid (t+1)$	$2 \le d \le \frac{q+1}{3}+t$	^36^
$s(q+1)$	$1 \le s \le q-1$	$2 \le d \le s$	^37^
$(\mu +1)\frac{q^2-1}{\gamma }$	$q\equiv -1~(\text {mod}~4)$, $\gamma \mid 2(q-1)$ and $\gamma \nmid (q-1)$, $0\le \mu \le \frac{\gamma }{4}-1$	$2\le d \le (\frac{\gamma +4}{8}+\mu )\frac{2(q-1)}{\gamma }+1$	Theorem 8
$(2\mu +1)\frac{q^2-1}{\gamma }$	$\gamma \equiv 2(\text {mod 4})$, $\gamma \mid (q+1)$, $0\le \mu \le \frac{\gamma -2}{4}$	$2 \le d \le \frac{q+1}{2}+(\mu +1)\frac{q+1}{\gamma }-1$	Theorem 12

## Data Availability

All data generated or analysed during this study are included in this published article.
